# Indirect Competitive Determination of Tetracycline Residue in Honey Using an Ultrasensitive Gold-Nanoparticle-Linked Aptamer Assay

**DOI:** 10.3390/molecules25092144

**Published:** 2020-05-04

**Authors:** Yan-Mei Sheng, Jian Liang, Jing Xie

**Affiliations:** 1 School of Pharmacy, Chengdu Medical College, Chengdu 610500, China; yanmeisheng@cmc.edu.cn; 2 Centre for Natural Products Research, Chengdu Institute of Biology, Chinese Academy of Sciences, Chengdu 610041, China; liangjian@cib.ac.cn

**Keywords:** tetracycline, honey, gold nanoparticle-linked aptamer assay

## Abstract

Tetracycline residue in honey has become an increasingly important food safety problem. In this work, an ultrasensitive gold nanoparticles (AuNPs)-linked aptamer assay was developed to determine the tetracycline residue in honey. First, a tetracycline–bovine serum albumin conjugate coating was applied to a microplate. Then, with the incubation of AuNPs-linked aptamer, the fixed tetracycline in the microplate competed for the limited aptamer with the free tetracycline in the sample. Higher amounts of free tetracycline in the sample were associated with more competitive binding of aptamer-AuNPs, and the aptamer-AuNPs binding with tetracycline-BSA was lower. Finally, as a kind of nanozyme, AuNPs exhibited peroxidase activity and oxidized 3,3′,5,5′-tetramethylbenzidine, transforming it from colorless to blue, and achieving the measurement at 652 nm. The analytical performance—including linearity, limit of detection, selectivity, precision, repeatability, and accuracy—has been investigated. It was successfully applied to the determination of tetracycline in honey samples with high accuracy and sensitivity.

## 1. Introduction

Honey is very popular as a natural nutritional tonic. However, the residual tetracycline in honey has become an increasingly important food safety problem. Tetracycline is widely approved for use in food-producing animals around the world with a certain tolerance dose [1]. Due to its potential toxic effects, the WHO, USA, EU, China, and other nations or organizations have specified a maximum tetracycline residue in honey of 0.1 ppm in order to protect consumer health. As a result, the precise and efficient detection of tetracycline residue in honey is of great significance.

To date, multiple tetracycline analysis methods have been established to date for this purpose. HPLC/UV [2,3,4,5,6] and HPLC/MS methods [7,8,9,10,11] have become the routine strategy because they give the best detection performance. However, an HPLC method based on the sample separation requires complicated pretreatment process, advanced professional technical skills, and relatively complicated instruments, which restricts its application to a large extent. Alternatively, in recent years, some new analysis methods based on immunology principles have been reported that have greatly simplified the pretreatment of samples, shortened the analysis time, and lowered the requirements of instruments [12,13,14,15,16,17,18,19]. These methods have mainly relied on the highly selective recognition of tetracycline antibody against tetracycline, and partial methods are still dependent on the signals based on enzyme-catalyzed reactions, such as horseradish peroxidase, which is the most commonly used enzyme in ELISA. As a biomacromolecule, the inherent limitations in terms of antibodies and enzymes are inevitable, e.g., difficult preparation, poor stability, poor repeatability for batches, high detection cost, etc.

The aptamer is the oligonucleotide sequence acquired through SELEX and it has an affinity and specificity that equal those of the monoclonal antibody. Moreover, an aptamer has the following advantages over an antibody: a sequence determined through in vitro screening and without immunogenicity and toxicity; chemical synthesis and labeling; good stability and invulnerability to solvent and temperature changes; short preparation period; low cost; and little variation among batches. There have been some reports on the tetracycline detection in food samples with aptamers [20,21,22,23,24,25,26,27,28]. However, there are still some shortcomings, such as complicated material fabrication, low sensitivity based on nanogold aggregation, the requirement of enzymes for color development, or the needed for special instruments. Nanomaterials have special and unexpected physical and chemical properties in an immunoassay [29,30,31]. Gold nanoparticles (AuNPs) were reported to have peroxidase activity and could catalyze hydrogen peroxide to generate a hydroxyl radical. The further reaction of a hydroxyl radical could change the colorless substrate to colored products, which is beneficial in terms of detection. AuNPs not only have better enzymatic activity than natural enzymes (such as HRP), but also have certain advantages over HRP, including low cost, stable catalytic activity, and convenience for large-scale preparation. Therefore, an AuNPs-linked aptamer could be applied to non-enzyme catalysis analysis based on the high selectivity of the aptamer for the analyte and the enhanced catalysis capacity of AuNPs [32,33,34].

In this work, tetracycline was coupled to BSA by a glutaraldehyde crosslinking method, and then applied to a microplate, for the purpose of the competitive binding with aptamer between fixed tetracycline in the microplate and free tetracycline in the sample. Simultaneously, tetracycline aptamer-labeled gold nanoparticles (aptamer-AuNPs) were synthesized. Then, a new competitive tetracycline detection method in honey was established based on the high selectivity of aptamer for the analyte and the enhanced catalysis capacity of AuNPs. The new method has various advantages, including easy realization of material preparation, high selectivity of analysis results, a low detection limit, and good stability of batches. It provides a new and feasible evaluation scheme for the detection of tetracycline residue in honey.

## 2. Results and Discussion

### 2.1. Principle of Analysis

**Figure 1:**
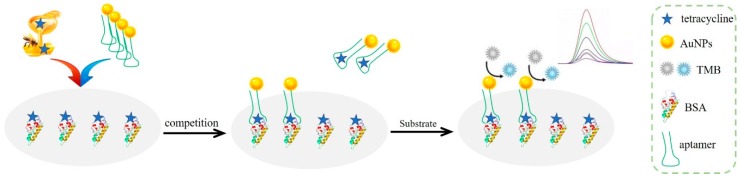
Illustration of the proposed gold nanoparticle-linked aptamer assay.

The analysis principle of the research was the indirect competition method, i.e., the analyte-protein conjugate was fixed, and the aptamer and free analyte were in the solution. The fixed analyte competed for the limited aptamer with the free analyte. Finally, the quantitative analysis was achieved through the color development of AuNPs catalysis substrate. The illustration of the proposed analytical procedure is shown in Figure 1. Firstly, the 5′-end sulfydryl modified tetracycline-aptamer-labeled gold nanoparticles (aptamer-AuNPs) were acquired by Au-S covalent bond, with the features of tetracycline recognition function and catalytic property of nanozyme. Then, tetracycline was fixed on BSA to generate the conjugate (tetracycline-BSA), which was subsequently used to coat the bottom of a microplate. The microplate is a high binding type with polystyrene as the carrier, which can improve the sensitivity, thus reducing the concentration and dosage of coating protein, but it is easy to produce non-specific adsorption, so another protein needs to be treated as a blocking agent. After blocking by casein solution, aptamer-AuNPs, and honey samples were added into the wells of a microplate. This enabled a competitive binding with the limited amount of aptamer between fixed tetracycline in the microplate and free tetracycline in the sample. The result of the competition is that aptamer-AuNPs bound with free tetracycline were removed, while aptamer-AuNPs bound with tetracycline-BSA were combined on the microplate. The horseradish peroxidase-hydrogen peroxide–3,3′,5,5′–tetramethylbenzidine (HRP-H_2_O_2_-TMB) colored system is commonly used in ELISA [35]. AuNPs were used to replace HRP in this work. AuNPs have been reported to have peroxidase activity and could catalyze hydrogen peroxide to a generate hydroxyl radical. Further reaction of hydroxyl radical could oxidize the colorless substrate (TMB) to its blue oxidized product, thus achieving the quantitative measurement at 652 nm. The peroxidase activity of AuNPs was obstructed to a certain extent due to the surface modification by the aptamer. Therefore, gold seed growth solution was used to generate gold shell sediments with larger surface area to obtain a better catalysis effect. Larger amounts of free tetracycline in the sample were associated with more competitive binding of aptamer-AuNPs, less aptamer-AuNPs binding with tetracycline-BSA, and a lighter final color. The depth of the color can be converted to a quantitative value by an ultraviolet spectrophotometer. As a result, the content of tetracycline in the sample was in negative correlation with the color depth.

### 2.2. Characterization of Tetracycline-BSA and Aptamer-AuNPs

**Figure 2:**
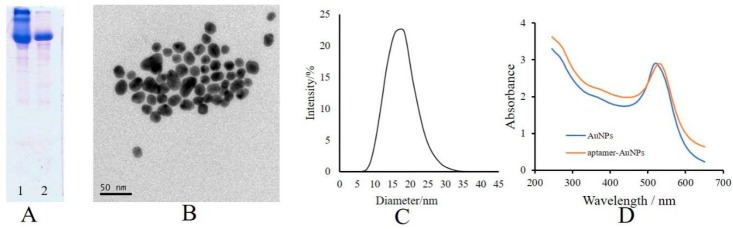
Results of material characterization: (**A**) SDS-PAGE of BSA and tetracycline-BSA; (**B**) TEM image of aptamer-AuNPs; (**C**) particle size distribution of aptamer-AuNPs; (**D**) UV–vis spectra of the AuNPs and aptamer-AuNPs.

In this work, the conjugate of tetracycline with bovine serum protein (tetracycline-BSA) was prepared by the glutaraldehyde crosslinking method. Glutaraldehyde is a bifunctional reagent with two aldehyde groups that can form a Schiff base with an amino group on the small molecule and the carrier protein, respectively. This method is widely applied for the preparation of coating antigens in an immunoassay because of its short reaction time in mild conditions, environmental protection, and ease of operation. The velocity of BSA was slightly greater than that of tetracycline-BSA in the SDS-PAGE analysis (Figure 2A), which proves the successful the crosslinking reaction of tetracycline and BSA. The result of transmission electron microscope (TEM) analysis is shown in Figure 2B. The result of dynamic light scattering (DLS) analysis is shown in Figure 2C with an average diameter of ~18 nm. The UV–vis spectra of the AuNPs and aptamer-AuNPs are shown in Figure 2D with maximum absorption peaks at 520 nm and 530 nm, respectively. The values for extinction coefficient of AuNPs at 450 nm and 520 nm are 1.39 × 10^8^ and 1.97 × 10^8^, respectively. The absorption value of AuNPs solution at 450 nm was 3.681; thus, the concentration of AuNPs solution was 9.512 nM according to Equation (1), which was equivalent to 5.75 × 10^15^ per mL converted by the Avogadro constant. Furthermore, the coupling ratio between the aptamer and AuNPs was obtained as follows: first, the concentrations of AuNPs solution and aptamer-AuNPs solution were diluted till to their absorption values at 520 nm (A_Au(520)_ and A_apt-Au(520)_), and this was assigned the value 1. Then, the absorption values of them at 260 nm (A_Au(260)_ and A_apt-Au(260)_) were determined to be 3.094 and 3.020. Thus, the coupling ratio was calculated as being about 52:1 according to Equation (2).

### 2.3. Analytical Performance of the Present Method for Tetracycline Determination

**Figure 3:**
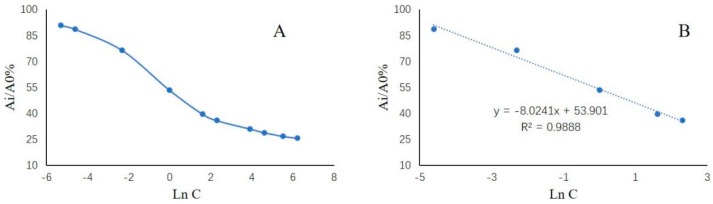
(**A**) Relative absorbance of the designed analytical method as a function of the logarithm of concentration. (**B**) Tetracycline standard curve.

The calibration curve was established using the logarithm of concentration (ln C, x) versus the ratio of the corresponding absorption values at 652 nm (A_i_/A_0_%_,_ y) as y = −8.024x + 53.901 with good correlation (r^2^ = 0.9888) in the range of 0.01–10 ng/mL (Figure 3). The LOD value for tetracycline was found to be 0.0027 ng/mL. Precision is an important indicator of an analytical method. This includes intra- and inter-day precision. Three batches of tetracycline quality control samples were tested with the spiked amounts of 0.1 ng/mL, 1 ng/mL, and 5 ng/mL, respectively. By measuring each concentration seven times, we calculated the mean detected amount and the coefficient of variation (CV), shown in Table 1, which are in accordance with the provisions of biological analysis. The results of six independent determinations with a spiked sample was 0.95 ± 0.05 ng/mL, which confirmed the good repeatability of the present method. The recovery measurements were performed to assess the accuracy of the gold nanoparticle-linked aptamer assay. The negative samples were spiked with three concentration levels of standard tetracycline. The results shown in Table 2 indicated that the recovery rates were in the range of 91.50–95.33% with CV < 5%. The selectivity was evaluated according to the influence that ten times as many possible interfering antibiotics as tetracycline had on the results of tetracycline determination. The results revealed that the detected content of tetracycline in the presence of various possible interfering substances was almost unchanged (the recovery of tetracycline was 92.54 ± 4.27%), indicating the good selectivity of the present method. In summary, the method validation results indicate that the gold nanoparticle-linked aptamer assay reached the quantitative analysis requirement and can be applied for the determination of tetracycline in honey. As shown in Table 3, the analytical figures of merit were compared with those of several other quantitative methods reported for tetracycline determination.

### 2.4. Detection of Tetracycline in Real Samples

A total of eight batches of honey samples purchased from a local supermarket was investigated by the proposed AuNPs-linked aptamer assay. Because honey is a weak acid, it can react slowly with metal. The galvanized iron bucket is a common container for storing honey. Thus, it is possible to greatly increase the content of zinc in honey. According to the national food safety standard (GB14963-2011), the limit of zinc in honey is 25 ppm. When developing the sample preparation methods for tetracycline, the high propensity for forming chelation complexes with divalent metal ions should be considered. Disodium ethylenediamine tetraacetate (Na_2_EDTA) has a greater affinity for the cations than tetracycline. Thus, Na_2_EDTA was used in sample pretreatment to achieve high recovery of tetracycline. In this study, tetracycline residue detected by the proposed method in five samples were negative, and in the other three samples, the detected amounts were 7 ng/g, 33 ng/g, and 38 ng/g respectively, all of which met the requirements of the national standards. These negative samples have been confirmed by HPLC/MS methods. One of them was randomly selected as the negative control sample for method validation. Obviously, this newly established method was able to analyze tetracycline content in honey with high accuracy and sensitivity.

## 3. Materials and Method

### 3.1. Reagents and Instruments

**Figure 4:**
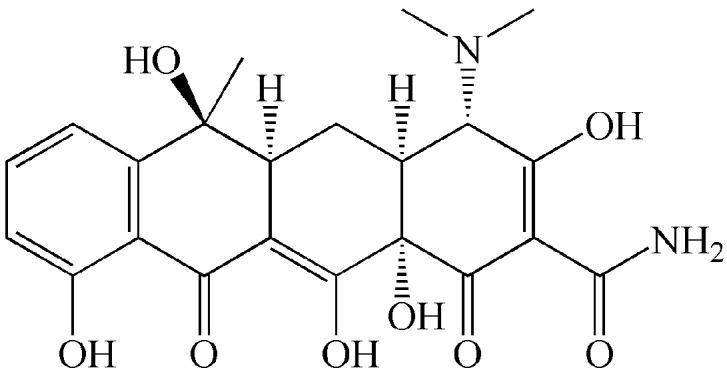
Chemical structure of tetracycline.

Chloroauric acid (HAuCl_4_), sodium citrate (Na_3_C_6_H_5_O_7_), disodium hydrogen phosphate dodecahydrate (Na_2_HPO_4_·12H_2_O), disodium ethylenediamine tetraacetate (Na_2_EDTA), Tween 20, tris(hydroxymethyl)aminomethane (Tris), hydrochloric acid (HCl), sodium chloride (NaCl), magnesium chloride (MgCl_2_), potassium chloride (KCl), calcium chloride (CaCl_2_), potassium dihydrogen phosphate (KH_2_PO_4_), hydroxylamine hydrochloride (NH_2_OH·HCl), tris(2-carboxyethyl)phosphine (TCEP), hydrogen peroxide (H_2_O_2_), 3,3′,5,5′-tetramethylbenzidine (TMB), and glutaraldehyde were purchased from Sinopharm Chemical Reagent Co., Ltd. (Shanghai, China). Tetracycline (CAS no. 60-54-8, Figure 4) and detachable 96-well microplate (FEP100008, JET) were provided by Shanghai Tansoole Technology Co. Ltd. (Shanghai, China). Bovine serum albumin (BSA) and casein were purchased from Shuangliu Zhenglong Biochemical Products Research Office (Chengdu, China). The dialysis bag was purchased from Viskase Corporation (Lombard, IL, USA). Tetracycline aptamer with 5′-end sulfydryl modified (Sequence: 5’-SH-(CH_2_)_6_-CGT ACG GAA TTC GCT AGC CCC CCG GCA GGC CAC GGC TTG GGT TGG TCC CAC TGC GCG TGG ATC CGA GCT CCA CGT G-3′, aggregate MW 23563.26) was provided by Sangon Technology Co., Ltd. (Shanghai, China). Honey samples and purified water (Wahaha Group Co., Ltd. Hangzhou, China) were purchased from local supermarket. The morphology and particle size were measured by FEI Tecnai G20 transmission electron microscope (FEI, Co., Ltd., Hillsboro, OR, USA). The test parameters of TEM are as follows: accelerating voltage 200 kV, point resolution 0.24 nm, information resolution 0.14 nm, electron gun energy resolution ≤ 0.7 eV, stem resolution > 0.2 nm. The UV absorbance values were determined by a UV–vis spectrophotometer (UV-L5S, Shanghai INESA Scientific Instrument Co., Ltd., Shanghai, China) and a Microplate Reader (PT 3502G) manufactured by Beijing Putianxinqiao Technology Co., Ltd. (Beijing, China).

### 3.2. Preparation of the Conjugate of Tetracycline with Bovine Serum Protein (Tetracycline-BSA)

The tetracycline-BSA conjugate was obtained by a glutaraldehyde crosslinking method following the procedures reported previously [36]. A brief description of steps is as follows: First, 20 mg tetracycline was dissolved in 3 mL pyridine and 40 mg BSA was dissolved in 5 mL PBS buffer (pH 7.4) solution. Then the tetracycline solution was added to the BSA solution with mild agitation. Subsequently, 50 μL of 25% glutaraldehyde solution was slowly dropped in. The solution was reacted at 4 °C and left overnight with slow stirring. Finally, a standard dialysis procedure was carried out for three days and the dialyzate was changed three times per day. Tetracycline-BSA was obtained after vacuum freeze drying, and it was stored in a refrigerator at 4 °C before use.

### 3.3. Preparation of the Tetracycline-BSA-Coated Microplate

A tetracycline-BSA solution dissolved in Tris-HCl buffer (10 mmol/L, pH 8.0) with a concentration of 4 μg/mL was poured into a 96-well microplate (100 μL in each well). After incubation for 10 h at 4 °C, the microplate was washed with 250 μL Tris-HCl-T buffer (10 mM, containing 0.05% Tween20, pH 7.6) three times. Then, 200 μL of 0.25% casein solution dissolved in Tris-HCl buffer (10 mmol/L, pH 8.0) was added to the wells, and the microplate was incubated at 37 °C for 30 min. Finally, after being washed by Tris-HCl-T buffer three times, the successful preparation of a tetracycline-BSA-coated microplate was complete, and it was stored in a refrigerator at 4 °C before use.

### 3.4. Preparation of Tetracycline Aptamer Labeled Gold Nanoparticles (Aptamer-AuNPs)

Firstly, a chemical reduction was carried out with sodium citrate as a reducing agent to obtain AuNPs as follows: First, 1 mL of 1% Na_3_C_6_H_5_O_7_ solution was quickly added to 10 mL of boiling 0.035% HAuCl_4_ solution. Secondly, heating and boiling was sustained until the solution turned wine-red for 5 min. Then it was cooled to room temperature. Finally, the AuNPs solution was successfully prepared by adding purified water into the solution up to 10 mL. The concentration of the resulting solution n can be calculated according to Equation (1)

(1) $$C=A_{450}/\varepsilon_{450}$$

where A_450_ is the absorption of AuNPs solution at 450 nm measured by a quartz cuvette (10 × 10 mm, id), and ε_450_ is the extinction coefficient of AuNPs at 450 nm.

Subsequently, 0.1 mL of 0.1 mM tetracycline aptamer solution dissolved in a 10 mM of Tris-HCl buffer solution activated by TCEP was added to the AuNPs solution obtained above. After being incubated for 16 h with electric-magnetic stirring at room temperature in the dark, 0.1 mL of 0.5 M Tris-acetate solution and 1 mL of 1 M NaCl solution were added to the mixture which was left for 24 h at room temperature in the dark. Finally, the precipitation was collected by centrifugation (16,000× *g*, 20 min), and washed twice with Tris-acetate/NaCl solution. After being re-dispersed, the tetracycline aptamer labeled gold nanoparticles (aptamer-AuNPs) were successfully prepared and it was stored in a refrigerator at 4 °C before use. The coupling ratio between aptamer and AuNPs can be calculated according to Equation (2) [34,37,38]

(2) $$\frac{n_{apt}}{n_{AuNps}}=\frac{C_{apt}}{C_{AuNps}}=\frac{(A_{apt-Au(260)}-A_{Au(260)})/\varepsilon_{apt(260)}}{A_{Au(520)}/\varepsilon_{Au(520)}}$$

where A*_apt-Au_*_(260)_ is the absorption value of aptamer-AuNPs at 260 nm, and A*_Au_*_(260)_ and A*_Au_*_(520)_ are the absorption values of AuNPs at 260 nm and at 520 nm, respectively. Each absorption value is measured by a standard quartz cuvette (10 × 10 mm, id). *ε_apt_*_(260)_ is the extinction coefficient of aptamer at 260 nm, *ε_Au_*_(520)_ is the extinction coefficient of a given particle size of AuNPs at 520 nm.

### 3.5. Determination of Tetracycline in Honey

Five grams of honey were dissolved in 20 mL of PBS-EDTA buffer solution (composed of 3.733 g Na_2_EDTA, 0.8 g NaCl, 0.02 g KCl, 0.29 g Na_2_HPO_4_·12H_2_O, 0.024 g KH_2_PO4, 100 mL H_2_O, pH 7.4). After 2 min of ultrasonic treatment, centrifugation (10,000× *g*, 10 min) was carried out. The supernatant was carefully transferred and diluted it 100 times with the aptamer binding buffer (composed of 20 mM Tris-HCl, 100 mM NaCl, 2 mM MgCl_2_, 5 mM KCl, 1 mM CaCl_2_, 0.02% Tween 20, pH 7.6) to create the test solution. Then, 50 μL of test solution and 50 μL of aptamer-AuNPs were added to the tetracycline-BSA-coated microplate (obtained in Section 2.3) and incubated for 30 min at room temperature with mild shaking. After being washed with 200 μL aptamer binding buffer three times, 100 μL of gold reinforcing agent (mixed by 5 mM HAuCl_4_·4H_2_O and 10 mM NH_2_OH·HCl in 1:1 proportion) was added and incubated for 2 min. The mixture was washed with 200 μL aptamer binding buffer three times. Finally, 100 μL of substrate solution (composed of 4.5 mL buffer (2.84 g Na_2_HPO_4_·12H_2_O, 1.92 g citric acid, 100 mL H_2_O), 0.5 mL of 2 mg/mL TMB solution, and 0.32 mL of 30% H_2_O_2_, well-mixed) was added. after incubation for 15 min, the absorbance value at 652 nm (A_652_) was measured. Throughout, the dilution factor was taken into account.

### 3.6. Validation Procedure

In this study, a negative honey sample without tetracycline confirmed by HPLC/MS was used for method validation. A_0_ and A*_i_* were defined as the absorbance values of negative control and samples at 652 nm, respectively, and their ratio of them (A_i_/A_0_%) was used for content calculation. The method was validated by evaluating the linearity, limit of detection (LOD), selectivity, precision, repeatability, and accuracy. The linearity of the method was demonstrated using a calibration standard of tetracycline. A calibration curve was prepared with tetracycline standards of five different concentrations (0.01 ng/mL, 0.1 ng/mL, 1 ng/mL, 5 ng/mL, and 10 ng/mL). The calibration curve was plotted by the logarithm of concentration (ln C, x) versus the ratio of A_i_/A_0_% (y). LOD was estimated as the concentration corresponding to three times of standard deviations below the mean absorbance from the negative control. The precision of the method was estimated by measuring the values of the mean, standard deviation, and coefficient of variation (CV) obtained by intra-day and inter-day determination of three concentrations of tetracycline spiked in the negative honey sample (0.1 ng/mL, 1 ng/mL, and 5 ng/mL). The repeatability of the method was estimated from six independent measurements of a spiked sample (1 ng/mL) analyzed under the experimental conditions. The accuracy of the method was evaluated based on the recoveries of tetracycline by spiking with tetracycline standard in a negative honey sample at three levels of 0.1 ng/mL, 1 ng/mL, and 5 ng/mL, then processing and measuring under the experimental conditions. The selectivity was evaluated by another recovery test of a spiked sample (1 ng/mL) with possible interfering antibiotics at 10 ng/mL, including chloramphenicol, aureomycin, sulfadiazine, furacilin, amoxicillin, gentamicin, and amikacin.

## 4. Conclusions

In this work, an ultrasensitive AuNPs-linked aptamer assay was developed to determine the tetracycline residues in honey. An aptamer can achieve specific recognition of tetracycline in the sample. AuNPs act as a kind of nanozyme exhibited peroxidase activity and oxidized 3,3′5,5′-tetramethylbenzidine for color system and signal enhancers. The analytical performance—including linearity, limit of detection, selectivity, precision, repeatability, and accuracy—have been investigated. This is the first report of an enzyme-free ELISA-like assay with easily to prepared materials, high specificity, low detection limit, low cost, and simple instrument requirements providing reliable detection of tetracycline residues in honey.

## Figures and Tables

**Table 1:** Results of precision evaluation.

Spike (ng/mL)	Intra-Day Precision		Inter-Day Precision	
	Detected (ng/mL)	CV	Detected (ng/mL)	CV
0.1	0.10 ± 0.00	3.46%	0.10 ± 0.01	7.17%
1	0.97 ± 0.03	3.11%	0.97 ± 0.05	5.20%
5	4.76 ± 0.12	2.55%	4.75 ± 0.20	4.25%

**Table 2:** Results of recovery evaluation.

	Spike (ng/mL)	Detected (ng/mL)	Recovery	Mean Recovery	SD	CV
1	0.1	0.0893	89.30%	91.50%	4.34%	4.74%
2	0.1	0.0965	96.50%			
3	0.1	0.0887	88.70%			
4	1	0.914	91.40%	93.00%	1.42%	1.52%
5	1	0.935	93.60%			
6	1	0.941	94.10%			
7	5	4.76	95.20%	95.33%	3.20%	3.36%
8	5	4.61	92.20%			
9	5	4.93	98.60%			

**Table 3:** Comparison of the analytical methods for tetracycline determination.

Method	Application	Apparatus	Range	LOD	Reference
SPR aptasensor containing oriented aptamer	honey	Biacore T200 SPR instrument	0.01–1000 μg/kg	0.0069 μg/kg	[20]
Enzyme-linked aptamer assay with multivalent HRP-mimicking DNAzyme	honey	UV–vis spectrophotometer	1.0 × 10^−2^ to 1.0 × 10^4^ ng/mL	8.1 × 10^−2^ ng/mL	[21]
direct competitive assay-based aptasensor	honey	microplate reader	0.1–1000 ng/mL	0.0978 ng/mL	[22]
Indirect competitive assay-based aptasensor	honey	microplate reader	0.01–100 ng/mL	9.6 × 10^−3^ ng/mL	[23]
electrochemical aptasensor based on poly (L-glutamic acid)/MWCNTs modified glassy carbon electrode	honey	potentiostat/galvanostat	1.0 × 10^−16^–1.0 × 10^−6^ M	3.7 × 10^−17^ M	[24]
Two aptasensors based on modified carbon paste/oleic acid and magnetic bar carbon paste/Fe3O4@oleic acid nanoparticle electrodes	drug, milk, honey and serum	potentiostat/galvanostat	1.0 × 10^−12^–1.0 × 10^−7^ M; 1.0 × 10^−10^–1.0 × 10^−7^ M;	3 × 10^−13^ M; 2.9 × 10^−11^ M;	[25]
Graphene oxide-based aptasensor	honey	UV–vis spectrophotometer	0.002–20 ng/mL	0.001 ng/mL	[26]
Colorimetric aptamer biosensor	milk	Microplate Spectrophotometer		122 nM	[27]
Colorimetric aptasensor	milk	UV–vis spectrophotometer	0.20–2.0 μg/mL	0.039 μg/mL	[28]
Electrochemical immunosensor	milk	electrochemical workstation	0.08–1 ng/mL	0.0321 ng/mL	[16]
Near-infrared fluorescence-based multiplex lateral flow immunoassay	milk	odyssey infrared imaging system	0.04–0.98 ng/mL	0.04 ng/mL	[15]
Immunochromatographic assay	serum	microplate reader	0.7–26 ng/mL	0.2 ng/mL	[14]
Aptamer-based magnetic solid-phase extraction	water and honey	HPLC/UV	10–3000 µg/L	2.5 µg/L	[6]
gold nanoparticle-linked aptamer assay	honey	microplate reader	0.01–10 ng/mL	0.0027 ng/mL	This work
